# Integration of long-read sequencing, DNA methylation and gene expression reveals heterogeneity in Y chromosome segment lengths in phenotypic males with 46,XX testicular disorder/difference of sex development

**DOI:** 10.1186/s13293-024-00654-8

**Published:** 2024-10-08

**Authors:** Agnethe Berglund, Emma B. Johannsen, Anne Skakkebæk, Simon Chang, Julia Rohayem, Sandra Laurentino, Arne Hørlyck, Simon O. Drue, Ebbe Norskov Bak, Jens Fedder, Frank Tüttelmann, Jörg Gromoll, Jesper Just, Claus H. Gravholt

**Affiliations:** 1 Department of Clinical Genetics, Aarhus University Hospital, Aarhus, Denmark; 2 Department of Molecular Medicine, Aarhus University Hospital, Aarhus, Denmark; 3 Department of Clinical Medicine, Aarhus University, Aarhus, Denmark; 4 Centre of Reproductive Medicine and Andrology, University of Münster, Münster, Germany; 5 Children’s Hospital of Eastern Switzerland, St. Gallen, Switzerland; 6 Department of Radiology, Aarhus University Hospital, Aarhus, Denmark; 7 Centre of Andrology & Fertility Clinic, Odense University Hospital, Odense, Denmark; 8 Centre of Medical Genetics, Institute of Reproductive Genetics, University of Münster, Münster, Germany; 9 Department of Endocrinology, Aarhus University Hospital, Aarhus, Denmark

**Keywords:** 46,XX testicular disorder/difference of sex development, Long-read sequencing, Sex development, Sex differentiation, Sex chromosomes, Disorders/differences of sex development.

## Abstract

**Background:**

46,XX testicular disorder/difference of sex development (46,XX DSD) is a rare congenital condition, characterized by a combination of the typical female sex chromosome constitution, 46,XX, and a variable male phenotype. In the majority of individuals with 46,XX DSD, a Y chromosome segment containing the sex-determining region gene (*SRY)* has been translocated to the paternal X chromosome. However, the precise genomic content of the translocated segment and the genome-wide effects remain elusive.

**Methods:**

We performed long-read DNA sequencing, RNA sequencing and DNA methylation analyses on blood samples from 46,XX DSD (*n* = 11), male controls (46,XY; variable cohort sizes) and female controls (46,XX; variable cohort sizes), in addition to RNA sequencing and DNA methylation analysis on blood samples from males with Klinefelter syndrome (47,XXY, *n* = 22). We also performed clinical measurements on all 46,XX DSD and a subset of 46,XY (*n* = 10).

**Results:**

We identified variation in the translocated Y chromosome segments, enabling subcategorization into 46,XX DSD (1) lacking Y chromosome material (*n* = 1), (2) with short Yp arms (breakpoint at 2.7–2.8 Mb, *n* = 2), (3) with medium Yp arms (breakpoint at 7.3 Mb, *n* = 1), and (4) with long Yp arms (*n* = 7), including deletions of *AMELY*,* TBLY1* and in some cases *PRKY*. We also identified variable expression of the X-Y homologues *PRKY* and *PRKX*. The Y-chromosomal transcriptome and methylome reflected the Y chromosome segment lengths, while changes to autosomal and X-chromosomal regions indicated global effects. Furthermore, transcriptional changes tentatively correlated with phenotypic traits of 46,XX DSD, including reduced height, lean mass and testicular size.

**Conclusion:**

This study refines our understanding of the genetic composition in 46,XX DSD, describing the translocated Y chromosome segment in more detail than previously and linking variability herein to genome-wide changes in the transcriptome and methylome.

**Supplementary Information:**

The online version contains supplementary material available at 10.1186/s13293-024-00654-8.

## Abstract (plain language summary)

Our sex chromosome constitution determines our sex, with the presence of a Y chromosome causing male sex determination (typically 46,XY) and the lack hereof resulting in female sex development (typically 46,XX). However, individuals can become male despite of them presenting with the classical female chromosome constitution. We term this “46,XX testicular disorder/difference of sex development” or “46,XX DSD” for short. Individuals with this condition are affected by a number of traits, such as infertility, short stature and altered hormone levels. We know that most cases of 46,XX DSD are caused by the sex-determining region of the Y chromosome being moved to one of the X chromosomes. However, we do not know the more precise size of the Y-chromosomal section or its effects on the genome. In this study we investigated 11 individuals with 46,XX DSD using novel and precise techniques than previously applied. Analyzing DNA, we found that their Y-chromosomal sections varied. This was reflected in multiple types of additional genetic analyses, investigating modifications to DNA and expression of genes. Our findings are the most accurate description of the Y-chromosomal section in 46,XX DSD to date, and indicate that the genomes of individuals with 46,XX DSD differ, with many potential mechanisms of action.

**Supplementary Information:**

The online version contains supplementary material available at 10.1186/s13293-024-00654-8.

## Highlights

• From a cohort of 11 individuals with 46,XX testicular DSD, the profile of Y chromosome segments was described in more detail than previously and used to sub-categorize subjects into four groups; *SRY*-negative, short Yp (2.7–2.8 Mb), medium Yp (7.3 Mb) and long Yp.
• In individuals with long Yp segments, a characteristic loss of *AMELY*,* TBL1Y* and in some cases *PRKY* was observed.
• Variable expression of the X-Y homologous gene pair *PRKX* and *PRKY* was observed, suggesting a compensatory mechanism.
• The lengths of Y chromosome segments were reflected in the transcriptome and methylome.
• Differential expression and methylation of autosomes suggested genome-wide effects.

**Supplementary Information:**

The online version contains supplementary material available at 10.1186/s13293-024-00654-8.

## Introduction

46,XX testicular disorder/difference of sex development (DSD) is a rare congenital condition which is clinically ascertained in approximately 3 per 100,000 newborn males [1]. Following the 2006 Chicago Consensus Statement on management of intersex disorders, 46,XX testicular DSD falls under the classification of “Disorders/Differences of Sex Development”, encompassing congenital conditions in which the chromosomal, gonadal and/or anatomical sex is atypical [2]. Here, when referring to males with 46,XX testicular DSD, the term “46,XX DSD” is utilized.

46,XX DSD are characterized by a combination of the typical female sex chromosome constitution 46,XX, testis formation and male differentiation of the embryonic Wolffian ducts in addition to fetal virilization of external genitalia. A complete male phenotype is typical, but various degrees of under-virilization with cryptorchidism, micropenis, and hypospadias are also observed [3]. In adulthood, small testes and hypergonadotropic hypogonadism with decreased testosterone levels and increased follicle-stimulating hormone (FSH) and luteinizing hormone (LH) levels are characteristic [4]. Thus, if untreated, affected individuals will experience the consequences of testosterone deficiency. Gynecomastia and short stature are other regularly reported traits [1, 4–6]. 46,XX DSD often remains undiagnosed for years, if ever diagnosed, with a median age at diagnosis of 25 years [1]. This fits well with infertility being the most common cause of ascertaining the diagnosis [1].

Human gonadal differentiation is a tightly coordinated spatiotemporal process. The bipotential gonads commit to either the testis- or ovary-specific pathway around week six of gestation. The major switch triggering the cascade of genetic events necessary for testis formation is up-regulation of the sex-determining region Y gene (*SRY)*, which is normally located on the short arm of the Y chromosome [7]. In more than 90% of cases of 46,XX DSD, a Y chromosome segment containing *SRY* has been translocated to typically the paternal X chromosome during meiosis, resulting in male differentiation [8]. *SRY* is located just below the pseudoautosomal region 1 (PAR1). Non-allelic homologous recombination between the protein kinase Y-linked gene *PRKY* and the X-linked *PRKX* on the X chromosome is thought to account for up to 70% of these translocations [9, 10]. Approximately 10% of 46,XX DSD do not present *SRY* (*SRY* negative 46,XX DSD). Instead, other aberrant genetic mechanisms such as up-regulation of *SOX9*, due to duplications in the upstream enhancer, or loss-of-function variants in genes related to the ovary-specific pathway (e.g. *RSPO1*), have been described [11, 12]. However, in the majority of these cases, the genetic mechanism are unknown [13].

X chromosome inactivation results in random silencing of one of the two X chromosomes in females and thereby balances gene expression between sexes [14]. However, in humans, about 20–30% of X-linked genes, most being located in the PAR1 of the X chromosome, have developed mechanisms to avoid silencing and therefore remain expressed from the inactive X [14]. It is not clear if X chromosome inactivation occurs similarly in 46,XX DSD and normal 46,XX females.

Although translocation of a Y chromosome segment containing *SRY* is the major underlying mechanism of 46,XX testicular DSD, the precise genomic content of the translocated segment, breakpoints and the effect on other biological pathways essential for the phenotype remains unknown. Males with Klinefelter syndrome (KS, 47,XXY) resemble 46,XX DSD in having an extra X chromosome, and in recent years, studies have shown that the altered X chromosome dosage in KS not only affects gene regulation on the sex chromosomes but indeed throughout the entire genome [15, 16], and candidate genes that may be implicated in the phenotype have been identified [15, 17, 18].

**Fig. 1:**
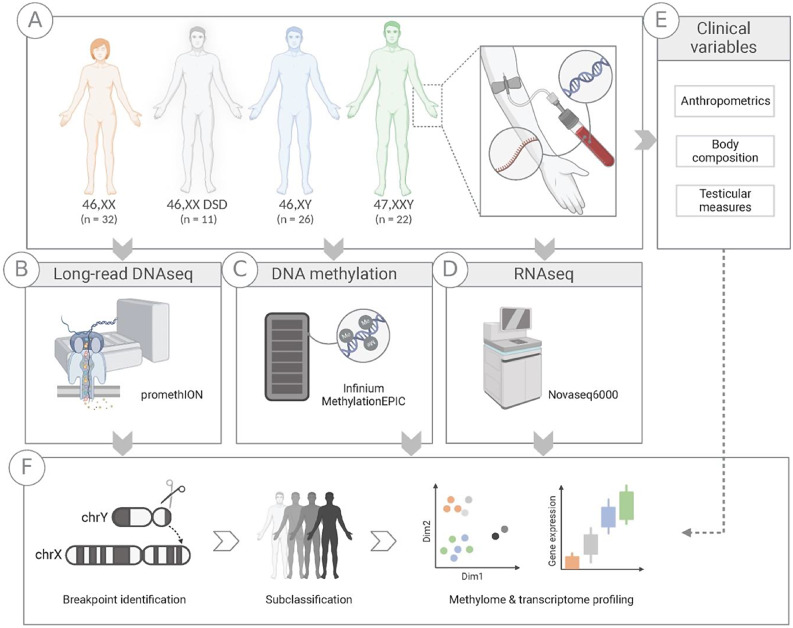
Study overview. Blood samples from males with 46,XX testicular DSD (46,XX DSD, *n* = 11), female controls (46,XX, *n* = 32), male controls (46,XY, *n* = 26) and males with Klinefelter syndrome (47,XXY, *n* = 22) (**A**) were used as starting material for DNA, RNA and methylation analysis (**B**-**D**). Purified DNA was used for long-read DNA sequencing (46,XX DSD, *n* = 11; 46,XX, *n* = 12; 46,XY, *n* = 12) (**B**) and DNA methylation analysis (**C**). Purified RNA was used for RNA sequencing to assess gene expression (**D**). In addition, clinical measurements were obtained from all 46,XX DSD and a subset of 46,XY males (*n* = 10), including anthropometrics, body composition and testicular measures (**E**). From the genetic data, 46,XX DSD were subclassified based on their Y chromosome profiles and breakpoint identification. Combined with the clinical measures, we describe the subgroups of the 46,XX DSD cohort (**F**)

To gain a comprehensive understanding of the molecular intricacies underlying 46,XX DSD, we performed an integrative approach combining long-read DNA sequencing, DNA methylation analysis, and gene expression profiling. By generating accurate reads up to 100 kb, long-read sequencing provides a unique opportunity to comprehensively characterize complex genomic regions and pinpoint the precise breakpoints of the translocated segment of the Y chromosome in l 46,XX DSD. Combined with DNA methylation analysis and gene expression profiling, this integrated analysis offers a unique opportunity to unravel the intricate interplay between genetic and epigenetic factors in 46,XX DSD and their implications for the phenotype compared to typical males (46,XY), females (46,XX) and males with KS (47,XXY) (Fig. 1).

## Methods

### Participants

The study included males with 46,XX DSD recruited from (1) the outpatient clinic at Department of Endocrinology and Internal Medicine, Aarhus University Hospital, and (2) the Fertility Clinic, Odense University Hospital. For comparison, male controls (46,XY, *n* = 26), female controls (46,XX, *n* = 32) and males with KS (47,XXY, *n* = 22) were included. Biological samples from female controls, KS participants, and male controls were available from previous studies of KS [15] and Turner syndrome [19]. Controls were recruited by advertisement in local newspapers and among previous participants in other trials. All participants had provided informed consent.

### Clinical data

Clinical data were collected for all 46,XX DSD and a subset of male controls (*n* = 10) (Fig. 1A, E).

#### Anthropometric measures

Anthropometric measures were height, weight, hip- and waist circumference. Measuring tape were used for arm span, hip- and waist circumference, all to the nearest millimeter. Body weight was measured to the nearest 0.1 kg. All measurements were performed by the same observer (AB). For 46,XX DSD, presence of gynecomastia and cryptorchidism were noted.

#### Body composition and bone mineral density

Total and regional fat mass, lean body mass and bone mineral density (BMD) were measured by dual-energy X-ray absorptiometry (DXA) using a Hologic 2000 (Hologic, USA). The system software provided the mass of lean body, fat and BMD for the whole body and specific regions. DXA was conducted in the morning after overnight fasting for at least 6 h.

#### Testicular measures

Scrotal ultrasonography was performed using a high-end ultrasound machine, GE Logiq E10, with linear probes, L6-15 and L2-9. Volumes were calculated as L x W x H x 0,71 (Lamberth) according to European guidelines [20]. Shear wave elastography was performed using L2-9 with 1 central ROI (small testes) and the results (m/s) were the median value of 10 cycles [21].

#### Statistics

Statistical comparisons of groups were analyzed by t-test or Mann-Whitney test as appropriate. All calculations were undertaken in StataCorp. 2021. Stata Statistical Software: Release 17. College Station, TX: StataCorp LLC.

### DNA preparation

Blood samples were drawn into EDTA treated tubes and stored immediately at -80° C. Genomic DNA was extracted using the QIAmp^®^ Mini Kit (Qiagen, Germany). The isolated DNA was quantified using a Qubit 4 fluorimeter (Thermo Fisher Scientific).

### Nanopore long-read sequencing

#### Sequencing and basecalling

Sequencing libraries were prepared according to the Nanopore protocols ‘Genomic DNA by Ligation (SQK-LSK110)’ version GDE_9108_v110_revL_10Now2020 and ‘Genomic DNA by Ligation (SQK-LSK114)’ version GDE_9161_v114_revH_29Jun2022 for 46,XX DSD and 46,XX and 46,XY controls, respectively, with an increased input of 1.5–2 µg of DNA and incubation times for End-prep at both 20 °C and 60 °C increased to 10 min. We used an input of 310 and 149 ng DNA for the loading mix for LSK110 and LSK114, respectively. To increase the output, we performed a flow cell wash and loaded the same libraries after both one and two days of sequencing. Nanopore sequencing was performed on PromethION 24. The sequencing produced between 51 and 135 Gb of raw data with a N50 of 16 to 29 kb per sample. The data was basecalled with Guppy version 6.1 and 6.3 for 46,XX DSD and 46,XX and 46,XY controls, respectively, with the high-accuracy model and filtering with a minimum Q-score of 9 (config = dna_r9.4.1_450bps_modbases_5hmc_5mc_cg_hac_prom).

#### Read mapping

Basecalled reads were mapped to a reference genome from the Telomere-to-Telomere consortium (T2T, chm13v2.0) [22] with minimap2 (v2.24), specifying ´-ax map-ont´ as recommended for multiple parameter settings for Oxford Nanopore reads. Additionally, ´--MD´ was included. Aligned reads were sorted with SAMtools (v1.15) and stored in BAM files. BAM files were filtered with a minimum quality score of 7 and a minimum length of 500.

#### Data visualization

Reads mapping to the X and Y chromosomes were loaded into R from BAM files with chromstaR (v1.22.0), and read densities were plotted with karyoploteR (v1.22.0). Data was also examined with the Integrative Genomics Viewer (IGV) (v2.16.0).

#### De novo assembly and sequence alignment

A local de novo assembly for reads mapping to the sex chromosomes was performed with Flye (v2.9), specifying --nano-hq and --meta. Sequences of genes of interest were downloaded from the UCSC Genome Browser. Using a standard nucleotide BLAST (blastn, BLAST v2.13.0), these sequences were aligned to the contigs generated from Flye.

### Infinium MethylationEPIC

1 µg of isolated DNA was bisulfide converted using the Illumina iScan platform. Methylation level was measured using the Infinium MethylationEPIC DNA Analysis BeadChip (Illumina) using the Illumina iScan platform at Eurofins Genomics AS.

Raw intensity values obtained from the Infinium MethylationEPIC chip was read into R (v. 4.1.1) and further processed using the R package Minfi. Cross reactive probes and poor performance probes with a detection p-value < 0.01 were filtered out. Next preprocessFunnorm normalization was applied; a between-array normalization method that removes variation by regressing out variability inferred by the control probes [23]. Cross reactive probes were then removed and the methylation values were calculated as M-values. Multidimensional scaling plots were evaluated to identify clusters of samples behaving differently than expected. Finally, probes were annotated to the human genome version 38 (R package: IlluminaHumanMethylationEPICanno.ilm10b5.hg38).

For differential methylation analysis, the M-values were analysed using LIMMA testing for differences at the group level, while adjusting for cell type abundance [24]. Cell type abundance estimation was carried out using EpiDISH. Differentially methylated positions were defined as adjusted p-value < 0.05 and delta-M > I1.0I.

DMRcate were used to identify DNA regions that were differentially methylated (DMRs), using a false discovery rate = 0.05, adjusted p-value < 0.05 and a |mean M-value| > 0.1. DMRs were defined as a minimum of two consecutive CpGs [25]. If more than 1000 nucleotides between significant CpGs, sites were divided into separate DMRs.

To investigate correlations between methylation and gene expression, all significant DMRs and differentially methylated positions (DMPs) (adj. P-value < 0.05) were correlated with gene expression, also including non-significant expressed genes, in order to evaluate trends in methylation status and gene expression. These were plotted in a scatter plot based on log2FC and delta-M.

#### Analysis of the presence of Y chromosome CpGs

Raw intensity values were analyzed by Ewastools [26]. In order to classify Y chromosome CpG probes as detected, a detection p-value was set at 0.01, and the number of detected Y CpGs was calculated for each subject and plotted according to karyotype.

### RNA sequencing

#### Sample preparation, library construction and sequencing

Blood samples for RNA extraction were drawn into PAXgene Blood RNA Tubes, placed at room temperature for two hours, and then sequentially stored at − 21 °C, before final storage at − 80 °C. The PAXgene blood Kit 262,174 (Qiagen) was used for purifying total-RNA. RNA quality was assessed by UV measurements on Lunatic (Unchained Labs) and on-chip electrophoresis on a Tapestation 4200 RNA Screen Tape System (Agilent, Denmark).

Synthesis of directional RNAseq libraries were conducted using the KAPA RNA HyperPrep with RiboErase Globin (HMR) (Roche, Denmark) according to recommended procedure. Library preparation was automated on a Sciclone NGS (Caliper, Perkin Elmer, Denmark) liquid handling robot. On-chip electrophoresis on Tapestation 4200 D100 Screen Tape System (Agilent) was used to estimate the quality of RNA-Seq libraries, and library concentrations were estimated using a Qubit dsDNA HS Assay (Thermo Fisher, Denmark). As input 500 ng RNA was used. RNA-seq libraries were multiplexed paired-end sequenced on Illumina Novaseq 6000 (100 bp).

#### RNAseq analysis

The raw fastq files were subjected to initial quality control using FastQC (Babraham Bioinformatics). Trimming of low-quality ends and adaptor removal was conducted using Trim Galore with default settings (Babraham Bioinformatics). Gene expression was quantified by quasi-mapping using Salmon [27]. A decoy-aware transcriptome index was built based on the hg38 transcriptome and selective alignment was run using the fastq pairs as input. Transcript abundancies were summarized to gene-level using the R package Tximeta [28]. Gene counts were then normalized using R Bioconductor package DESeq2 [29]. Only genes with at least 20 counts in more than 6 samples were included in the differential expression analysis. Statistical significance was denoted as a Benjamini-Hochberg adjusted p-value < 0.05.

#### Weighted correlation network analysis

To identify co-expressed genes from the RNA-seq data and associate these to cohort group- and clinical measures, weighted correlation network analysis (WGCNA, v1.71) was applied. A signed co-expression network was constructed using a one-step approach. Adjacency was calculated choosing an appropriate soft thresholding power with approximate scale-free topology. Gene clustering was performed on the signed Topology Overlap Matrix by hierarchical clustering, identifying modules via the blockwiseModules function. The module eigengenes were calculated with the moduleEigengenes function. For each module-trait association, eigengene significance and corresponding p-value were obtained. Intramodular connectivity and gene significance was extracted for each module of interest.

## Results

### Participants

The study included 46,XX DSD (*n* = 11) with a mean age of 44.2 [SD: ±13.0] years. For comparisons we included: male controls (46,XY, *n* = 26; mean age: 42.8 [SD: ±12.0]), female controls (46,XX, *n* = 32; mean age: 44.1 [SD: ±12.4]), and males with KS (47,XXY, *n* = 22; mean age: 40.2 [SD: ±7.9]) (Fig. 1A). There was no significant age difference between 46,XX DSD and 46,XY (*p* = 0.8), 46,XX (*p* = 0.9) or 47,XXY (*p* = 0.6).

### Clinical characterization

46,XX DSD had significantly lower height and weight than 46,XY, but there was no difference in body mass index (BMI), nor in hip- and waist circumference. Total body and total abdominal lean mass were also significantly lower in 46,XX DSD compared to 46,XY, whereas there was no difference in total body and total abdominal fat mass. Testicular height, width, and length were all considerably reduced in 46,XX DSD compared to 46,XY, resulting in a significantly lower testis volume (Table 1).

**Table 1:** Anthropometrics, blood pressure, body composition, bone mineral density, and testicular size in males 46,XX DSD and controls

		46,XX DSD (*n* = 11)	Male controls (*n* = 10)	*P*-value
Anthropometrics	Height (cm)	167.1 (161.5-181.6)	182.2 (160.8-188.1)	0.006*
	Weight (kg)	74.4 (65.4–88.6)	90.6 (47.4-107.1)	0.03*
	BMI (kg/m2)	26.4 ± 2.7	26.1 ± 4.3	0.9
	Hip (cm)	98.6 (91.4–111.0)	102.8 (83.0-110.0)	0.2
	Waist (cm)	92.0 ± 9.0	94.2 ± 11.6	0.6
Ambulatory blood pressure	Day systolic AMBP	123.8 ± 12.0	126.4 ± 9.2	0.6
	Night systolic AMBP	109.0 (93–151)	112.0 (96–129)	0.7
	24-h systolic AMBP	120.1 ± 13.0	123.9 ± 8.1	0.5
	Day diastolic AMBP	77.8 ± 8.3	79.0 ± 8.8	0.8
	Night diastolic AMBP	64.3 ± 11.3	67.5 ± 9.4	0.5
	24-h diastolic AMBP	74.4 ± 9.2	77.0 ± 7.5	0.5
Dual-energy X-ray absorptiometry	BMD lumbar spine (g/cm2)	0.97 (0.64–1.23)	0.97 (0.82–1.48)	0.8
	BMD hip (g/cm2)	1.2 ± 0.2	1.3 ± 0.2	0.4
	Total body fat (kg)	26.0 ± 7.1	26.3 ± 8.2	0.9
	Abdominal fat (kg)	12.8 ± 3.7	12.3 ± 4.1	0.8
	Abdominal lean body mass (kg)	23.0 (21.5–28.6)	29.7 (16.7–34.0)	0.01*
	Abdominal fat (%)	34.6 (20-43.2)	29.5 (21.4–63.5)	0.1
	Visceral fat (g)	583.8 ± 188.5	558.4 ± 168.8	0.8
	Total body lean mass (kg)	46.5 (43.5–59.0)	61.1 (33.9–68.3)	0.008*
	Total body fat (%)	29.6 (21.4–46.0)	33.2 (22.5–41.5)	0.1
Testis	Length (cm)	1.9 ± 0.3	4.4 ± 0.4	< 0.0001*
	Width (cm)	1.2 ± 0.2	2.8 ± 0.3	< 0.0001*
	Height (cm)	0.9 ± 0.2	2.4 ± 0.3	< 0.0001*
	Volume (ml)	1.6 ± 0.4	20.9 ± 6.1	< 0.0001*
	Elastography, right	1.2 (0.97–1.9)	1.3 (0.8–1.5)	0.5
	Elastography, left	1.3 (0.9-3.0)	1.2 (0.8–1.5)	0.5

### Y chromosome segmental heterogeneity in 46,XX DSD identified by long-read DNA sequencing

**Fig. 2:**
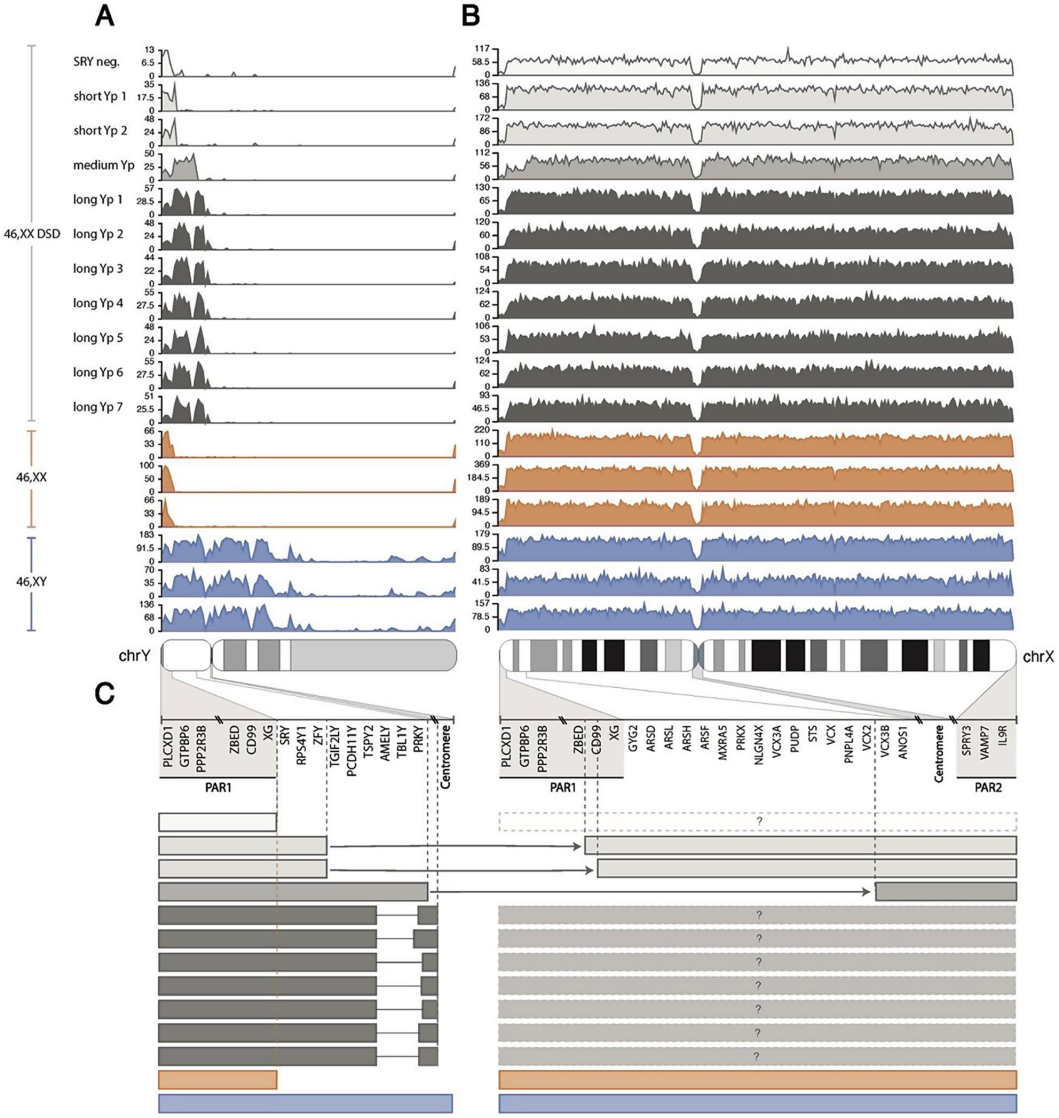
Y-chromosomal heterogeneity in 46, XX DSD identified by long-read DNA sequencing. Based on read densities across the Y (**A**) and X (**B**) chromosomes for 11 46,XX DSD, three representative female controls (46,XX; orange) and three representative male controls (46,XY; blue), we identified Y-chromosomal heterogeneity among 46,XX DSD within our cohort. The 11 46,XX DSD were separated into four groups according to Y-chromosomal segment length; *SRY* negative (*n* = 1; white), short Yp (*n* = 2; light grey), medium Yp (*n* = 1; dark grey) and long Yp (*n* = 7; black) (**A**). No obvious differences were observed from the read density across the X chromosome, except for a decreased read density in the distal part of Xp for the 46,XX DSD individual with a medium Yp segment (**B**). A schematic illustration of the X-Y translocations is presented in (**C**). As expected, the *SRY* negative 46,XX DSD individual only had Y-chromosomal reads spanning PAR1, similar to observations for females. The 46,XX DSD with short Yp segments had breakpoints after *ZFY* (chrY: 2,782 kb; chrY: 2,823 kb) on the Y chromosome, continuing upstream of *CD99P1* (chrX: 2,191 kb) and within *CD99* (chrX: 2,349 kb) on the X chromosome. The 46,XX DSD individual with a medium Yp segment had a breakpoint after *PRKY* (chrY: 7,275 kb) on the Y chromosome, continuing upstream of *VCX3B* (chrX: 8,018 kb) on the X chromosome, where read density increased in (**B**). Identification of breakpoints for the seven 46,XX DSD with longer Yp segments was not possible, however, they had reads spanning most of Yp all the way up to the centromere. When no knowledge of X-linked breakpoints was observed the boxes were marked with “?”

While previous studies have identified approximate breakpoints, the exact genomic content of the translocated Y chromosome segment and thereby genetic mechanisms underlying male sex differentiation among 46,XX DSD have not been established [30–33]. Therefore, we initially focused on evaluating the genetic material of Y-chromosomal origin in each 46,XX DSD individual with the application of long-read DNA sequencing (Oxford Nanopore). The read density of all reads mapping to the Y chromosome revealed heterogeneity (Fig. 2A). In some cases, breakpoints could be identified as multiple reads aligned to both the Y chromosome and X chromosome (split reads) (Supplemental Fig. 1B, C, Table 2). One individual with 46,XX DSD had reads restricted to the pseudoautosomal region 1 (PAR1), a 2.7 Mb region homologous between the sex chromosomes located distal on the p-arms, thus lacking Y-specific material, and thereby the *SRY* gene (referred to as “*SRY* negative”, *n* = 1). For this individual, no other genetic cause for the male sex development was identified. *SRY* was present in all other 46,XX DSD in the cohort (*SRY* positive) (*n* = 10). Two individuals with 46,XX DSD had reads limited to the distal part of Yp (referred to as “short Yp” 1 and 2, *n* = 2), with breakpoints on the Y chromosome situated distal of *ZFY* (short Yp 1: chrY:2,823 kb; short Yp2: chrY:2,782 kb) while breakpoints on the X chromosome were either proximal of *CD99P1* (short Yp 1: chrX:2,191 kb) or within *CD99* (short Yp 2: chrX:2,349 kb), both within PAR1 (Fig. 2C). One individual with 46,XX DSD had reads spanning about 75% of Yp (referred to as “medium Yp”, *n* = 1), with breakpoints identified distal of *PRKY* on the Y chromosome (chrY:7,275 kb) and between *VCX2* and *VCX3B* on the X chromosome (chrX:8,018 kb) (Fig. 2B), which also have Y-linked homologues. The remaining 46,XX DSD had reads spanning most of Yp (referred to as “long Yp” 1–7, *n* = 7) (Fig. 2; Table 2). Repetitive DNA sequences in the proximal part of Yp, especially around *FAM197Y-* and *TSPY-*genes, complicated the alignment, and thereby identification of breakpoints in these cases. However, data strongly indicated that all had breakpoints close to the centromere, distal of the *TSPY*-genes (Supplemental Fig. 2). Thus, 46,XX DSD could be sub-categorized into “*SRY* negative”, “short Yp”, “medium Yp”, and “long Yp” based on the presence and length of the translocated Yp segments, with breakpoints situated outside of the most proximal part of Yp in *SRY*-positive individuals.

A characteristic loss of Y chromosome material two thirds into Yp were observed for all “long Yp” individuals (Supplemental Fig. 1A-B) (discussed in further detail below). We did not find similar patterns of lost read density on the X chromosome in regions with X-Y homologues (Fig. 2B).

**Table 2:** Subgroups, X-Y breakpoints, *PRKY*-status and *PRKY/PRKX/ARSD* expression of 46,XX DSD

Subgroup		Breakpoints	PRKY (+/-)	PRKY expression	PRKX expression	ARSD expression
*SRY* neg.		NA	-	18.52	1608.70	375.8782
Short Yp	1	chrY:2,782 kb chrX:2,349 kb	-	0.00	1394.27	306.6739
	2	chrY:2,823 kb chrX:2,191 kb	-	0.00	1031.34	556.7005
Medium Yp		chrY:7,275 kb chrX:8,018 kb	+	1405.23	613.86	193.2793
Long Yp	1	NA	+	739.15	501.53	100.6634
	2	NA	+	1048.79	690.56	196.5956
	3	NA	-	0.00	1155.03	194.3472
	4	NA	+/-	120.32	1296.50	115.6697
	5	NA	-	0.34	1451.36	258.3894
	6	NA	+/-	170.39	937.00	170.4093
	7	NA	+/-	74.37	987.50	184.1210

### Y-chromosomal methylation and gene expression patterns

To support our findings, we employed gene expression profiling (RNAseq) and DNA methylation analysis of approximately 850,000 CpG sites (Infinium MethylationEPIC). CpG sites are regions in the DNA sequence comprising cytosine followed by a guanine nucleotide in the 5’ to 3’ direction, across the genome. These analyses compared 46,XX DSD to 46,XX, 46,XY, and 47,XXY.

**Fig. 3:**
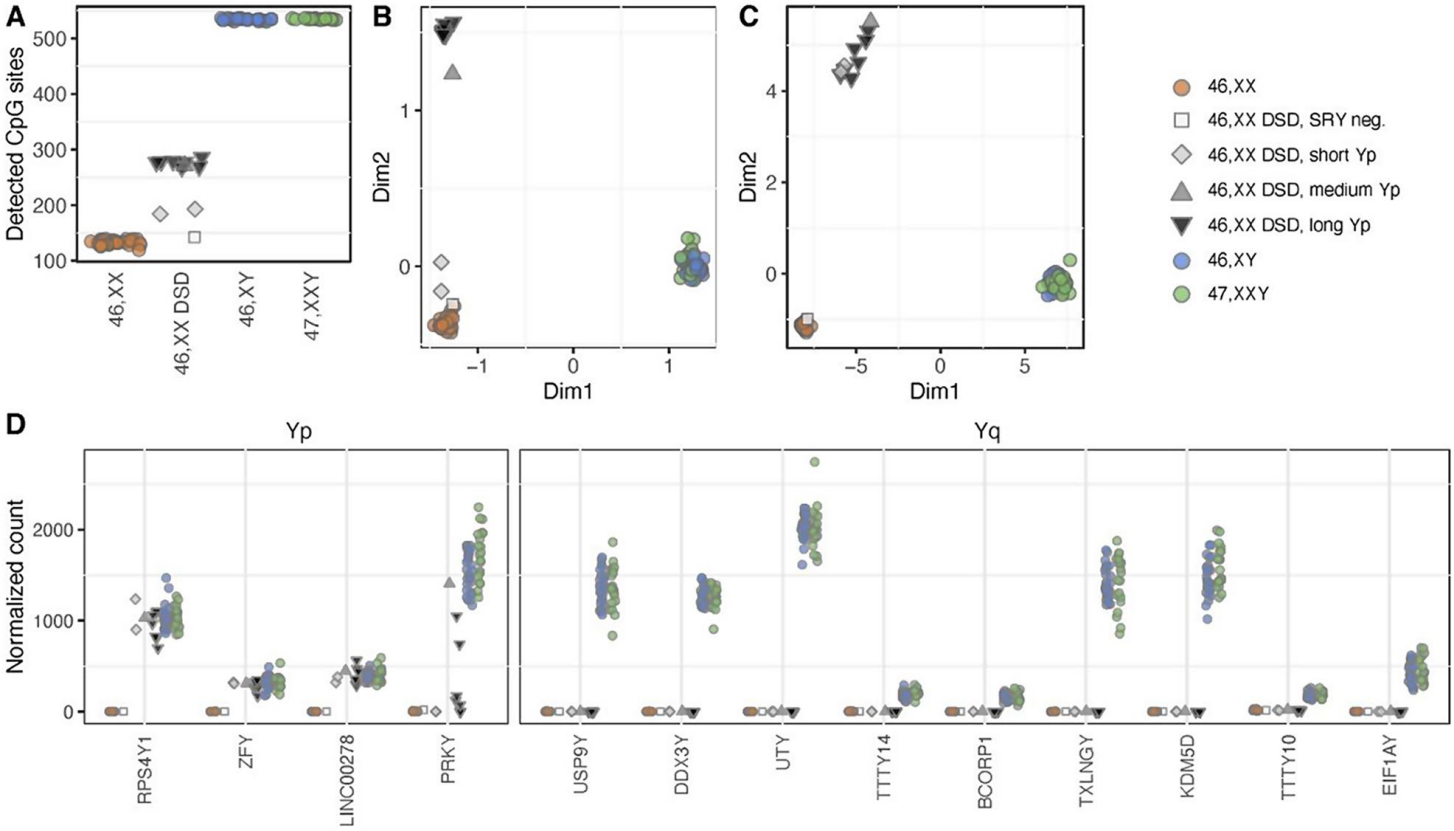
Y-chromosomal methylation and gene expression patterns. DNA methylation and gene expression data from the Y chromosome for all included groups. The number of Y-linked CPG-sites detected from DNA methylation data were denoted for each group. 46,XX DSD had in-between numbers of CpG sites between the numbers for 46,XX and 46,XY/47,XXY, with the CpG counts for each 46,XX DSD reflecting their Y chromosome segment lengths (**A**). Multi-dimensional scaling analysis of CpG sites from the Y chromosome showed the same in-between pattern of 46,XX DSD – depending on Y chromosome segment length (**B**). Principal component analysis of gene expression data from the Y chromosome showed that all 46,XX DSD with Y chromosome material clustered by themselves, except for the SRY negative 46,XX DSD clustering with 46,XX (**C**). Normalized counts of differentially expressed Y-chromosomal genes, sorted by position on the Y chromosome and separated into Yp and Yq, showed that *ZFY*, *RPS4Y1* and *LINC00278* expression was present in all 46,XX DSD with Y chromosome material. In the medium and long Y groups a variable expression of *PRKY* was observed (**D**). Colors indicate karyotype (46,XX: orange; 46,XY: blue; 47,XXY: green) and Y chromosome segment length for 46,XX DSD (SRY neg.: white; short Yp: light grey; medium Yp: dark grey; long Yp: black)

When evaluating CpG sites located on the Y chromosome, we found an average of approximately 130 Y-linked CpG sites for 46,XX (unspecific binding) and approximately 530 CpG sites for both 46,XY and 47,XXY, while 46,XX DSD had a heterogenic in-between number of CpG sites, corresponding to the length of the translocated Yp segment (Fig. 3A). Clustering based on the Y-chromosomal methylome also revealed that 46,XX DSD clustered according to the amount of translocated Y chromosome – some in a separate group (long Yp, medium Yp) and others in closer proximity to 46,XX (*SRY* negative, short Yp) (Fig. 3B).

Y-chromosomal gene expression showed that 46,XX DSD clustered separately from all other groups, except for the one *SRY* negative 46,XX DSD, who clustered with 46,XX (Fig. 3C). Among 46,XX DSD, four Y-chromosomal differentially expressed genes (DEGs) were upregulated compared to 46,XX; *ZFY*, *PRKY*, *RPS4Y1* and *LINC00278*, and 13 DEGs were downregulated compared to 46,XY and 47,XXY (Table 3, Supplementary File 1). When annotating the location of these genes to Yp and Yq, we identified a marked upregulation of Yp-genes compared to 46,XX and an absence of Yq-genes (Fig. 3D), supporting our finding that Y-chromosomal sequences in 46,XX DSD were restricted to Yp.

Thus, both the Y-chromosomal methylome and transcriptome supported our finding of Y-chromosomal segment heterogeneity among 46,XX DSD.

**Table 3:** Differentially expressed genes (DEGs)

Gene expression			
	Upregulated	Downregulated	Total
*Autosomes*			
46,XX DSD vs. 46,XX	27	21	48
46,XX DSD vs. 46,XY	24	40	64
46,XX DSD vs. 47,XXY	19	12	31
46,XX vs. 46,XY	539	486	1025
*X chromosome*			
46,XX DSD vs. 46,XX	1	3	4
46,XX DSD vs. 46,XY	22	2	24
46,XX DSD vs. 47,XXY	1	12	13
46,XX vs. 46,XY	45	20	65
*Y chromosome*			
46,XX DSD vs. 46,XX	4	0	4
46,XX DSD vs. 47,XXY	0	13	13

### Loss of Y chromosome segment spanning X-Y homologous regions

**Fig. 4:**
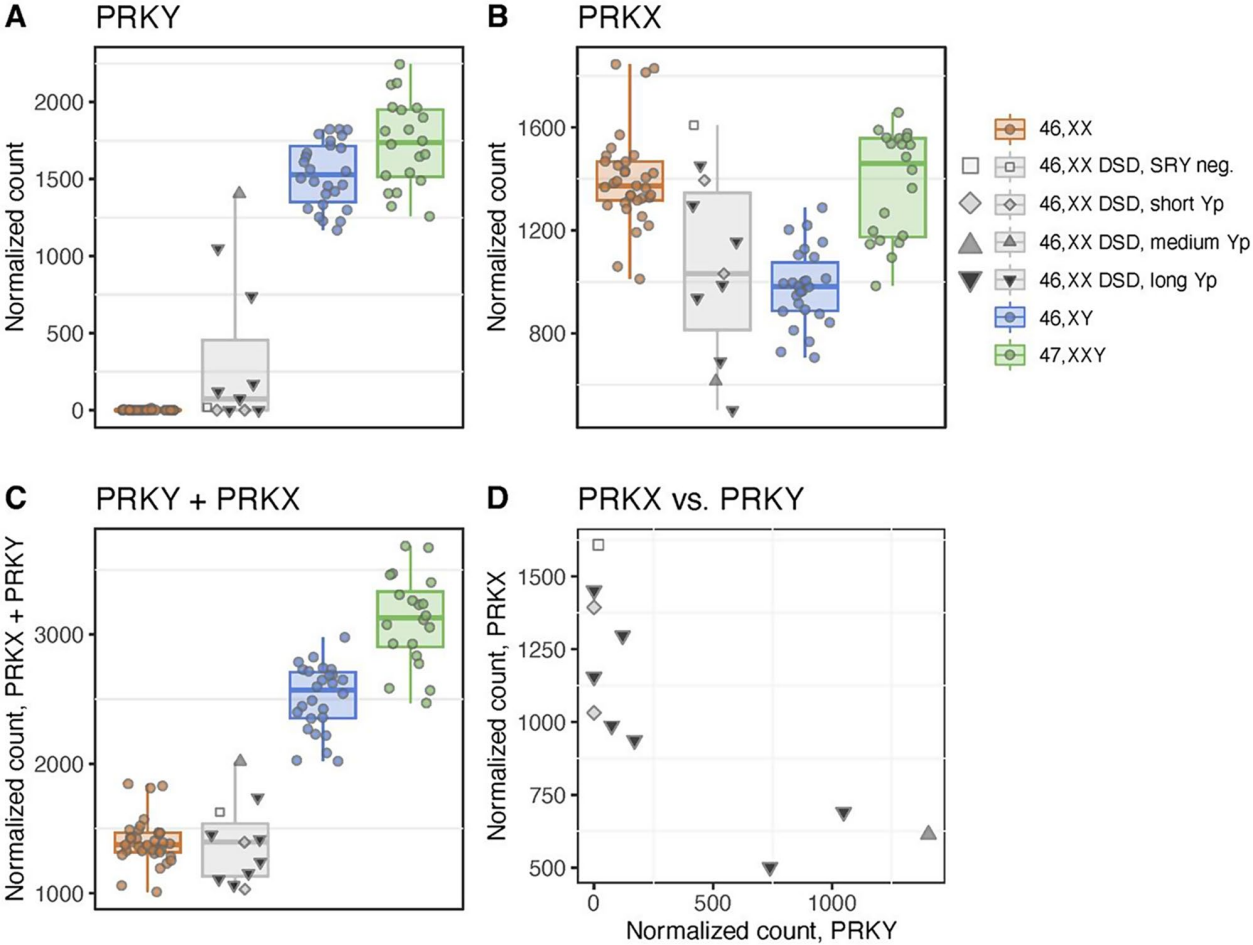
Variable expression patterns in the X-Y homologous gene pair *PRKY/PRKX*. Normalized expression of X-Y homologous gene pair *PRKY* (**A**) and *PRKX* (**B**), illustrating a variable expression pattern in 46,XX DSD. The summed gene expression of *PRKY* and *PRKX* showed expression levels close to those of 46,XX (**C**). *PRKY* expression vs. *PRKX* expression indicated that high *PRKY* expression led to lower *PRKX* expression in 46,XX DSD (**D**). Colors indicate karyotype (46,XX: orange; 46,XY: blue; 47,XXY: green) and Y chromosome segment length for 46,XX DSD (SRY neg.: white; short Yp: light grey; medium Yp: dark grey; long Yp: black)

As mentioned above, a specific part of Yp was missing in all “long Yp” 46,XX DSD. This region contained three genes with homologues on the X chromosome; *AMELY*, *TBLY1* and *PRKY*. We found a complete loss of both *AMELY* and *TBLY1*, but a variable partial loss of *PRKY* (1 x *PRKY*+, 2 x *PRKY*-, 4 x *PRKY*+/-) (Table 2, Supplemental Fig. 1C). The X-linked homologues to these genes, *AMELX*,* TBLX1 and PRKX*, had not been lost, but we found that 46,XX DSD had either mono- or biallelic partial *PRKX* deletions (Supplemental Fig. 3). Due to the variable *PRKY* patterns, we made an investigation of this region in respect to gene expression. According to their *PRKY-*patterns, some 46,XX DSD lacked *PRKY* expression, while others had levels close to those of 46,XY and 47,XXY (Fig. 4A; Table 2). Similar variation was observed for the X-linked homologue, *PRKX* (Fig. 4B). In general, we found no difference in the combined expression of *PRKY* and *PRKX* compared to 46,XX (Fig. 4C), however, we observed a higher expression of *PRKX* in 46,XX DSD lacking *PRKY* and vice versa, suggesting a compensatory mechanism and/or dysfunctional recombination between *PRKY* and *PRKX* (Fig. 4D). Based on this, we further sub-divided the 46,XX DSD into “*PRKY* high” and “*PRKY* low“ (Table 2, discussed in further detail below).

In conclusion, 46,XX DSD with longer Y-segments had a loss of specific Y-linked genes with X-linked homologues: *AMELY* and *TBLY1.* In addition, we observed a variable DNA loss and expression of *PRKY*, seemingly balanced by a variable expression of *PRKX.*

### The parentage of PAR1

Next, we aimed to identify the parentage of PAR1 on the X-Y translocated chromosome. First, a *de novo* assembly of the sex chromosomes was made for each individual with 46,XX DSD. Second, sex-chromosomal gene sequences were aligned to the contigs arising from the *de novo* assembly. The majority (7/8) had PAR1 genes and the beginning of *XG* and the *XG* pseudogene *XGY2* localized in the same contig. The continuation of the *XG* gene and subsequent X-linked genes were found in another contig, and the continuation of *XGY2* and the subsequent Y genes in a third (Supplementary Fig. 4). This indicated that PAR1 of the X chromosome, containing Y material in 46,XX DSD, mainly originates from the paternal Y, as previously shown [34].

### X-chromosomal and autosomal methylation and gene expression

**Fig. 5:**
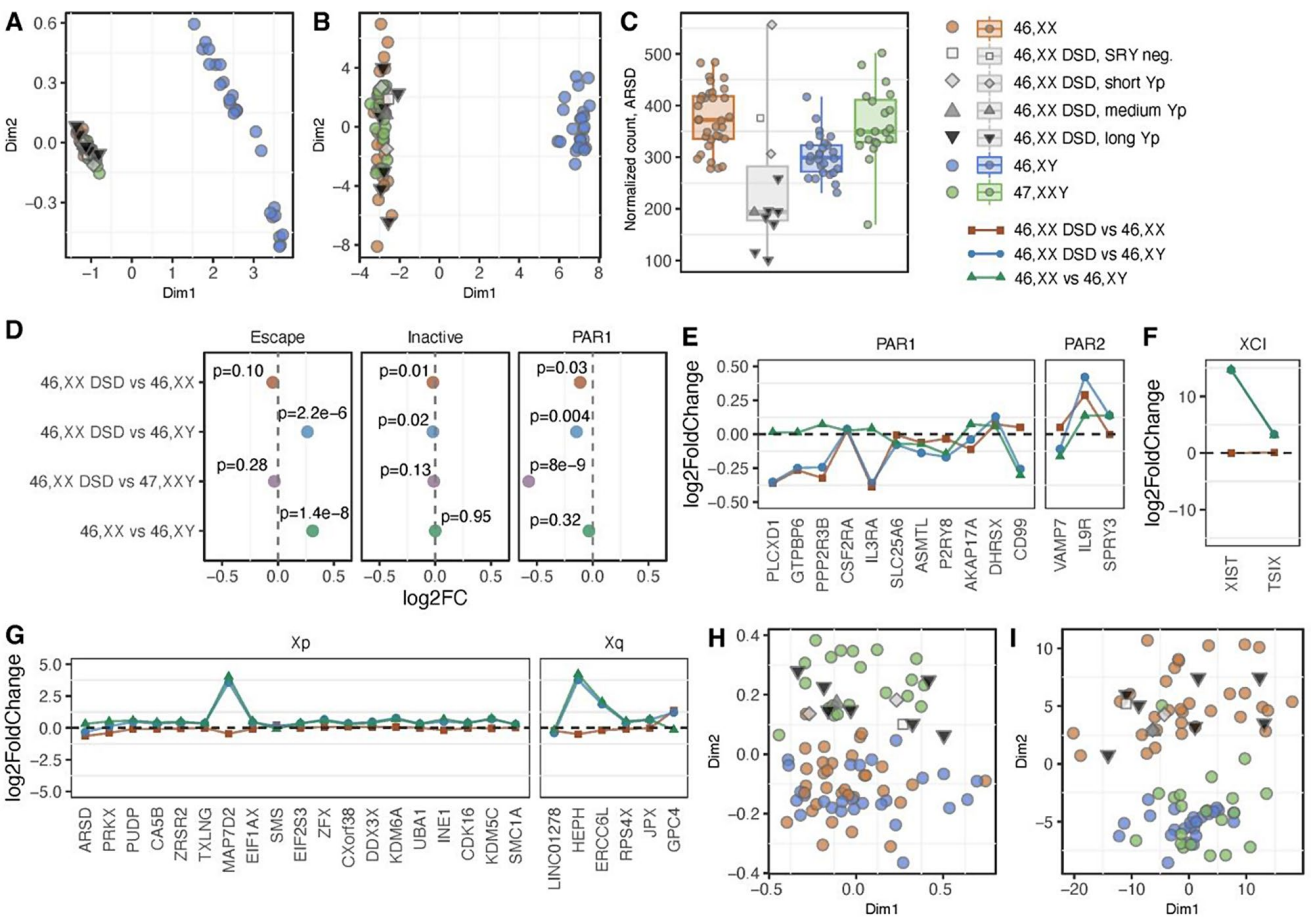
Autosomal and X-chromosomal methylation and gene expression patterns in 46,XX DSD. Multi-dimensional scaling analysis of DNA methylation (**A**) and principal component analysis of gene expression (**B**) from the X chromosome comparing 46,XX DSD, 46,XX, 46,XY and 47,XXY. Expression of the *ARSD* gene located on the X chromosome, which was differentially expressed in 46,XX DSD compared to both 46,XX and 46,XY. Here we observe a marked decrease in 46,XX DSD with medium or long Yp segments (**C**).The overall Log2FoldChange in X-chromosomal gene expression of all expressed escape genes, PAR1 genes and inactivated genes between 46,XX DSD and 46,XX, 46,XY and 47,XXY, as well as 46,XX and 46,XY (**D**). The individual gene expression changes between 46,XX DSD and 46,XX (red), 46,XX DSD and 46,XY (blue) and 46,XX and 46,XY (green) for genes expressed from PAR1 and PAR2 (**E**), X chromosome Inactivation (XCI) genes (**F**) and differentially expressed genes (padj < 0.05) in either 46,XX DSD vs. 46,XX or 46,XX DSD vs. 46,XY from the X chromosome (**G**). For panels E-G, the order of the genes was annotated according to chromosomal location (p to q arm). Multi-dimensional scaling analysis of DNA methylation (**H**) and principal component analysis of gene expression (**I**) from autosomes comparing 46,XX DSD, 46,XX, 46,XY and 47,XXY. Colors indicate karyotype (46,XX, orange; 46,XY, blue; 47,XXY, green) and Y chromosome segment length for 46,XX DSD (SRY neg.: white; short Yp: light grey; medium Yp: dark grey; long Yp: black)

As expected, cluster analysis based on the X-chromosomal methylome and the X-chromosomal transcriptome showed that individuals clustered according to the number of X chromosomes; 46,XX DSD, 47,XXY, and 46,XX clustered together, with 46,XY in a separate cluster (Fig. 5A, B)(Tables 4 and 5; Supplementary Files 2, 3). Differential methylation analysis of individual CpG sites (DMPs, deltaM > |1|, padj < 0.05) identified a single DMP that was specific for 46,XX DSD, situated in the promoter region of the transcription factor *SOX3*. We then identified differentially expressed genes (DEGs) (p adj < 0.05) (Table 3, Supplementary File 1). Only one X-chromosomal gene, *ARSD*, was differentially expressed in 46,XX DSD compared to both 46,XX and 46,XY. Expression of this gene was markedly downregulated in the “long Yp” 46,XX DSD compared to both control groups (Fig. 5C). This was in line with a previous study where the breakpoint of the X chromosome was found in the vicinity of the *ARSE* gene [30], located just downstream of *ARSD*.

**Table 4:** Differentially methylated positions (DMPs)

Differentially methylated positions (DMPs)			
	Hypermethylated	Hypomethylated	Total
*DMPs on autosomes*			
46,XX DSD vs. 46,XX	165	76	241
46,XX DSD vs. 46,XY	235	56	291
46,XX DSD vs. 47,XXY	22	17	39
46,XX vs. 46,XY	41	34	75
*DMPs on the X chromosome*			
46,XX DSD vs. 46,XX	2	0	2
46,XX DSD vs. 46,XY	5680	2560	8240
46,XX DSD vs. 47,XXY	5	1	6
46,XX vs. 46,XY	5608	2473	8081

**Table 5:** Differentially methylated regions (DMRs)

Differentially methylated regions (DMRs)			
	Hypermethylated	Hypomethylated	Total
*DMRs on autosomes*			
46,XX DSD vs. 46,XX	38	6	44
46,XX DSD vs. 46,XY	37	8	41
46,XX DSD vs. 47,XXY	5	2	7
46,XX vs. 46,XY	2	1	3
*DMRs on the X chromosome*			
46,XX DSD vs. 46,XX	0	0	0
46,XX DSD vs. 46,XY	661	297	958
46,XX DSD vs. 47,XXY	0	0	0
46,XX vs. 46,XY	655	286	941

We annotated X-chromosomal genes as escape, inactivated, and PAR, and compared changes in overall expression. As expected, escape genes had a higher level of expression in 46,XX DSD compared to 46,XY (*p* = 2.2e-6) and in 46,XX compared to 46,XY (*p* = 1.4e-8) (Fig. 5D). The overall expression of PAR1 genes was decreased in 46,XX DSD compared to both 46,XX (*p* = 0.03) and 46,XY (*p* = 0.004). This suggests that the expression of PAR1 genes derived from a Y chromosome may decrease when translocated to an inactive X chromosome, or that reduced expression could result from partial spreading of X-inactivation as described by Tukiainen et al. [35]. The same applied to inactive genes, where 46,XX DSD had a slightly decreased expression compared to both 46,XX (*p* = 0.01) and 46,XY (*p* = 0.02). The decreased expression of PAR1 genes in 46,XX DSD was clearly observed when plotting the individual expressional changes for all expressed PAR1 genes (Fig. 5E). The most notable expression changes were observed near the end of the p-arm of the X chromosome; however, this may simply reflect variability in the expression of PAR1 genes. An upregulation of *XIST* in 46,XX DSD compared to 46,XY was also observed (Fig. 5F) and X-linked DEGs were generally upregulated in this contrast as well (Fig. 5G).

We further investigated autosomal DNA methylation and gene expression. Cluster analysis of autosomal DNA methylation showed clustering of 46,XX DSD with 47,XXY, and of 46,XX with 46,XY (Fig. 5H). Furthermore, autosomal hypermethylation was observed in 46,XX DSD (Table 4, Supplementary File 2), supporting the finding of the clustering analyses (Fig. 5A). Forty-nine autosomal DMPs were unique to 46,XX DSD when compared to both 46,XX and 46,XY. Extending the analysis to the regional level (Table 5, Supplementary File 3), four DMRs were specific to 46,XX DSD, located in the genes *GFOD2*, *RASAL2*, *UNC5D* and *EBF4*.

Cluster analysis of autosomal gene expression showed clustering of 46,XX DSD with 46,XX, and 47,XXY with 46,XY (Fig. 5I), the latter in accordance with previous reports [34]. Three autosomal DEGs were unique for 46,XX DSD compared to both 46,XX and 46,XY; *LFNG*, *KLHL24*, and *C7orf26 (INTS15).*

Finally, X-chromosomal and autosomal DEGs were used as input for pathway enrichment analysis. This showed enrichment within DSDs (Turner syndrome, gonadal dysfunction, spermatogenic failure, and 46,XX DSD) and biological processes related to DNA methylation (Supplemental Fig. 5).

### Characterization of 46,XX DSD subgroups related to PRKY

To identify gene networks associated with 46,XX DSD, their subgroups and clinical traits, we performed WGCNA on gene expression data from 46,XX DSD and 46,XY. Here, co-expressed genes were separated into groups of so-called modules. Each module was assigned a unique color and correlated to karyotype and expression of *PRKY* and *PRKX.* We found that the lightcyan module was positively correlated to 46,XX DSD (0.93, *p* = 1e-09) and negatively to 46,XY (-0.93, *p* = 1e-09) (Supplemental Fig. 6), meaning that the majority of the genes within this module was overexpressed in 46,XX DSD. This module had a strong negative correlation to expression of *PRKY* (-0.85, *p* = 1e-06), but not to expression of *PRKX* (0.17, *p* = 0.5). When correlating the aforementioned module to clinical traits, we found an association to height (-0.57, *p* = 0.007), lean mass (-0.48, *p* = 0.03) and testicular measurements (width (-0.89, *p* = 8e-08), length (-0.89, *p* = 7e-08), height (-0.86, *p* = 5e-07), volume (-0.85, *p* = 1e-06)). Grouping 46,XX DSD by *PRKY* expression (low, 0-170; high, 739–1405 normalized counts), low expression was highly correlated to the lightcyan module as well (0.76, *p* = 7e-05). In contrast, high *PRKY* expression was not correlated to this module (0.28, *p* = 0.2). This could indicate that low *PRKY* expression in 46,XX DSD is associated with lower testis measures, height and lean mass, compared to 46,XX DSD with higher *PRKY* expression. Further studies are needed to validate this.

## Discussion

Using a multi-modal and multi-omics strategy implementing long-read DNA sequencing, DNA methylation, and gene expression, we show that 46,XX DSD can be divided into at least four different subgroups based on the size and pattern of the Y-chromosomal translocation. We also show that in the majority of the present cohort, a large segment of Yp has been translocated to the X chromosome. In addition, PAR1 on the translocated X chromosome is most likely of Y-chromosomal origin, meaning that a substantial proportion of Yp is present in most cases. We show that all four subtypes of 46,XX DSD have a specific autosomal and X-chromosomal DNA methylation and gene expression profile, indicating that the translocated Yp segment imposes global genomic changes. These changes correlated to phenotypic traits known to be present among 46,XX DSD, such as lower height, lean mass and testis size.

Previous studies investigating the genomics of 46,XX DSD have used techniques that have been unable to describe the exact breakpoints of the translocated Yp in detail. Most studies have utilized probes on the Y chromosome, not providing exact genomic locations. An early study from 1986 employed a panel using less than ten probes on Yp [36]. More recent studies performed Chromosomal Microarray Analysis (CMA), with a sensitivity based largely on the number of probes [9, 30]. Although these approaches provide much higher resolution than karyotyping and fluorescence in-situ hybridization (FISH) techniques, most CMAs are not designed to investigate large parts of the Y chromosome. Therefore, we decided to use long read sequencing to enable a more precise and comprehensive analysis of the complex genomic regions of 46,XX DSD. Its ability to generate reads up to 100 kb allowed for a precise characterization of the chromosomal rearrangements in our cohort. Furthermore, we used the recently published T2T genome as reference [22, 37], leading to a more precise alignment, and thus, delineation of chromosomal breakpoints in some instances.

Recombination can take place between the X and Y chromosomes in the homologous subtelomeric PARs, with PAR1 encompassing 2.7 Mb and PAR2 330 Kb [38]. The recombination rate in the male PAR1 is 17-fold elevated compared with the genome average, which is the highest rate in the genome, while the recombination rate in the female PAR1 is only slightly elevated [39]. In male meiosis, a crossover in PAR1 is required to ensure normal disjunction of the X and Y chromosomes, and loss of PAR1 leads to male sterility [40]. Thus, PAR1 is a male-specific recombination hotspot, while PAR2 is not. It has also become evident that PAR1 on the X and Y chromosomes show significantly different allele frequencies, which may imply that recombination does not sufficiently homogenize the gene pool present in the PAR regions [41].

Previously, it has been shown that non-allelic homologous recombination between the X and Y homologues *PRKX/Y*, situated just outside PAR1 and a hotspot for recombination, could explain most cases of 46,XX DSD [9, 10]. *PRKX* and *PRKY* are not completely identical, *PRKY* being slightly shorter than *PRKX* [10]. This difference is likely due to lack of recombination during the evolution of the two sex chromosomes [42]. A recent report of 20 46,XX DSD described varying degrees of Y-chromosomal material taking part in the translocation and at least eight different breakpoints were observed [30]. However, in that study, the methodology used did not allow a more precise characterization of the PAR1 region. The authors showed that nine in 20 cases had a breakpoint in *PRKX*, but they did not identify any with breakpoints close to the *PRKY* gene [30]. Our study extends these observations and shows that events leading to most cases of 46 XX testicular DSD are more complicated than hitherto thought. We show that indeed some 46,XX DSD are missing *PRKY*, and in addition all except one (“medium Yp”) missed *AMELY*, which also has an X homologue, *AMELX*. Based on a population study, few males are known to have a deletion of this gene together with *PRKY* and *TBL1Y* [43]. It has been suggested that descendants of males with a deleted *AMELY* are protected against 46,XX testicular DSD, as this region should be sponsoring non-allelic homologous recombination [10, 43]. Surprisingly, regions of the Y chromosome proximal to *PRKY* and *AMELY* were present among the “long Yp” 46,XX DSD, indicating a translocation process, where the entire Y PAR1 region, and neighboring genes including *SRY*, but not *AMELY* (and in some cases not *PRKY*), in addition to other Y genes more proximal, are translocated to the X chromosome are retained. While recent research has reported deletion of *ARSL* just downstream of X chromosome PAR1 in multiple 46,XX DSD [30], we found no decline in read density in any subjects using long-read sequencing.

The autosomal DNA methylation pattern among 46,XX DSD resembled the pattern previously observed in 47,XXY [15, 44]. Several genes with possible relevance for the 46,XX DSD phenotype showed differential methylation or expression specific to 46,XX DSD. The X-linked DMP *SOX3* has been linked to X-linked intellectual disability and 46,XX DSD [45–47]. Autosomal DMRs specific to 46,XX DSD were located in genes (*GFOD2*, *RASAL2*, *UNC5D*, *EBF4*) involved in neural development, netrin-receptor activation, neural migration and GTPase activation [48–51] and autosomal DEGs specific to 46,XX DSD (*LFNG*, *KLHL24* and *C7orf26/INTS15*) have been shown to be involved in Notch signaling during embryonic development, skin filament stability and regulation of RNA polymerase II [52–54].

The overall expression of PAR1 genes was decreased in 46,XX DSD compared to both 46,XX and 46,XY, most pronounced in the proximal part of PAR1. This was also the case for inactive genes, indicating that the translocation affects expression of genes closest to the breakpoint, consistent with other recent data showing that most genes within 100 kb of a breakpoint do show significant expression changes [55]. Here, we conclude that the fraction of genes with altered expression is dependent on the distance to a breakpoint.

We were previously able to link the clinical phenotype in 46,XX DSD with the gene expression changes, especially height [4], but also testicular measurements, likely due to the missing *AZF* genes. This indicates that genes in the translocated Y segment, or that the positional effect of the translocation itself, influences height [56]. Thus, the global changes induced by the length of translocations among 46,XX DSD could likely affect the phenotype. In addition, 46,XX DSD with low *PRKY* expression had a stronger correlation to a 46,XX DSD gene expression pattern than those with high *PRKY* expression. Position effects could also be due to changes in 3D genome structure, affecting the transcription both in *cis* and in *trans*, caused by the translocation disrupting coding sequences or non-coding genomic regions, either on the translocated part stemming from the Y chromosome, or the host X chromosome [56, 57].

## Conclusion

In conclusion, the translocation of Y-chromosomal material to the X chromosome in 46,XX DSD is complex, arising from at least four different scenarios. It includes translocation of the entire Y-chromosomal PAR1, in addition to varying Yp segments, including *SRY*. The translocation affects methylation and transcription globally, altering methylation level and transcription of several autosomal genes. The translocation further affects transcription in *cis* on the derivative X chromosome.

## Electronic supplementary material

Below is the link to the electronic supplementary material.

**Supplementary Material 1: Supplementary File 1.** Differential gene expression. Differential gene expression from autosomes, the X chromosome and the Y chromosome for contrasts 46,XX DSD vs, 46,XY; 46,XX DSD vs 46,XY; 46,XX DSD vs 47,XXY; and 46,XX vs 46,XY.

**Supplementary Material 2: Supplementary File 2.** Differentially methylated positions, Differentially methylated positions from the autosomes and the X chromosome for contrasts 46,XX DSD vs, 46,XY; 46,XX DSD vs 46,XY; 46,XX DSD vs 47,XXY; and 46,XX vs 46,XY.

**Supplementary Material 3: Supplementary File 3.** Differentially methylated regions. Differentially methylated regions from the autosomes and the X chromosome for contrasts 46,XX DSD vs, 46,XY; 46,XX DSD vs 46,XY; 46,XX DSD vs 47,XXY; and 46,XX vs 46,XY.

**Supplementary Material 4: Supplemental Fig. 1** Breakpoints on the Y chromosome. (A-C) Reads mapping to the Y chromosome in selected regions. A) One 46,XX DSD individual had reads restricted to PAR1 and lacked chromosome Y-specific material (SRY neg., panel 1). Breakpoints were identified for two 46,XX DSD (short Yp, panel 2–3) at 2,782 kb and 2,823 kb. B-C) One individual with 46,XX DSD had a breakpoint after PRKY (medium Yp, panel 1). In the area upstream, the same individual had a smaller (ca. 250 kb) deletion, whereas the remaining seven 46,XX DSD (long Yp, panel 2–8) had a larger deletion, spanning across *AMELY* and *TBL1Y* into *PRKY*. Picture from The Integrative Genomics Viewer (IGV).

**Supplementary Material 5: Supplemental Fig. 2** Incomplete alignment in highly repeated region near the centromere of the Y chromosome. A-B) The seven 46,XX DSD with longer Y chromosome segments (long Yp) had incomplete alignment in the region of the Y chromosome containing highly repeated sequences, including *TSPY*-genes and *FAM197Y*-genes. Reads continue to map to the Y chromosome until shortly before the centromere (B). Picture from The Integrative Genomics Viewer (IGV).

**Supplementary Material 6: Supplemental Fig. 3** Partial deletion in PRKX. A partial deletion in *PRKY* is found in 46,XX DSD (medium Yp, panel 1; representative long Yp, panel 2–4). Two representative 46,XY controls are presented in panel 5–6. Picture from The Integrative Genomics Viewer (IGV).

**Supplementary Material 7: Supplemental Fig. 4** Parentage of PAR1. A representative illustration of the Y chromosome segment in relation to PAR1 in *SRY*-positive 46,XX DSD based on de novo assembly and sequence alignments of the assembled contigs indicated that PAR1 is of parental origin.

**Supplementary Material 8: Supplemental Fig. 5** Differential expression analysis and gene enrichment analysis. Differentially expressed genes in the contrasts. 46,XX DSD vs 46,XX and 46,XX DSD vs 46,XY were used as input for gene enrichment analysis, respectively (Gene-Disease associations, Gene Ontology Biological processes (GOBP). DEGs from the 46,XX DSD vs 46,XY comparison were enriched in diseases related to disorders/differences of sex development and enriched in biological processes involved in methylation (A,B). DEGs from the 46,XX DSD vs 46,XX comparison were also enriched in diseases related to disorders/differences of sex development and enriched in one biological process related to the establishment of sister chromatids (C,D).

**Supplementary Material 9: Supplemental Fig. 6** Weighted Correlation Network Analysis (WGCNA). With gene expression from 11 46,XX DSD and 10 46,XY males as input, a Weighted Correlation Network Analysis (WGCNA) was run to identify modules of co-expressed genes and relate these to the following traits; Patient groups, testis measurements, anthropometrics & mass, bone and blood pressure. Correlation and p-values are shown for each module-trait correlation.

## Data Availability

Whole-genome long-read DNA sequencing, DNA methylation and RNA sequencing data are available at the European Genome-phenome Archive (EGA).
