# Supplementary figures and images for: Integration of long-read sequencing, DNA methylation and gene expression reveals heterogeneity in Y chromosome segment lengths in phenotypic males with 46,XX testicular disorder/difference of sex development
Source: Biol Sex Differ. 2024 Oct 8;15:77. doi: 10.1186/s13293-024-00654-8 (PMC11463111; doi:10.1186/s13293-024-00654-8)

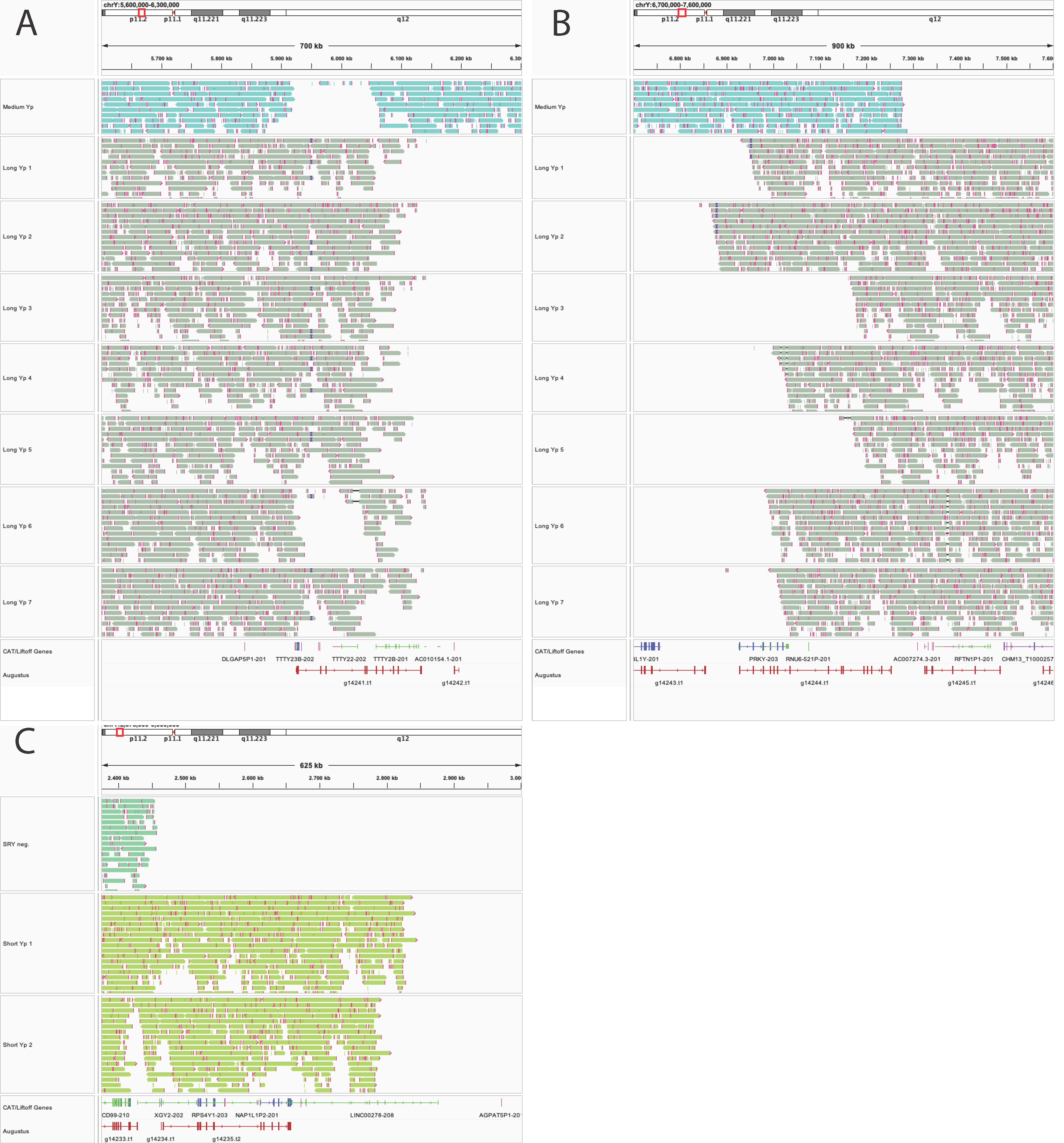

Supplement: Supplementary file 4 — Supplementary Material 4: Supplemental Fig. 1 Breakpoints on the Y chromosome. (A-C) Reads mapping to the Y chromosome in selected regions. A) One 46,XX DSD individual had reads restricted to PAR1 and lacked chromosome Y-specific material (SRY neg., panel 1). Breakpoints were identified for two 46,XX DSD (short Yp, panel 2–3) at 2,782 kb and 2,823 kb. B-C) One individual with 46,XX DSD had a breakpoint after PRKY (medium Yp, panel 1). In the area upstream, the same individual had a smaller (ca. 250 kb) deletion, whereas the remaining seven 46,XX DSD (long Yp, panel 2–8) had a larger deletion, spanning across AMELY and TBL1Y into PRKY. Picture from The Integrative Genomics Viewer (IGV). [file 13293_2024_654_MOESM4_ESM.jpg]

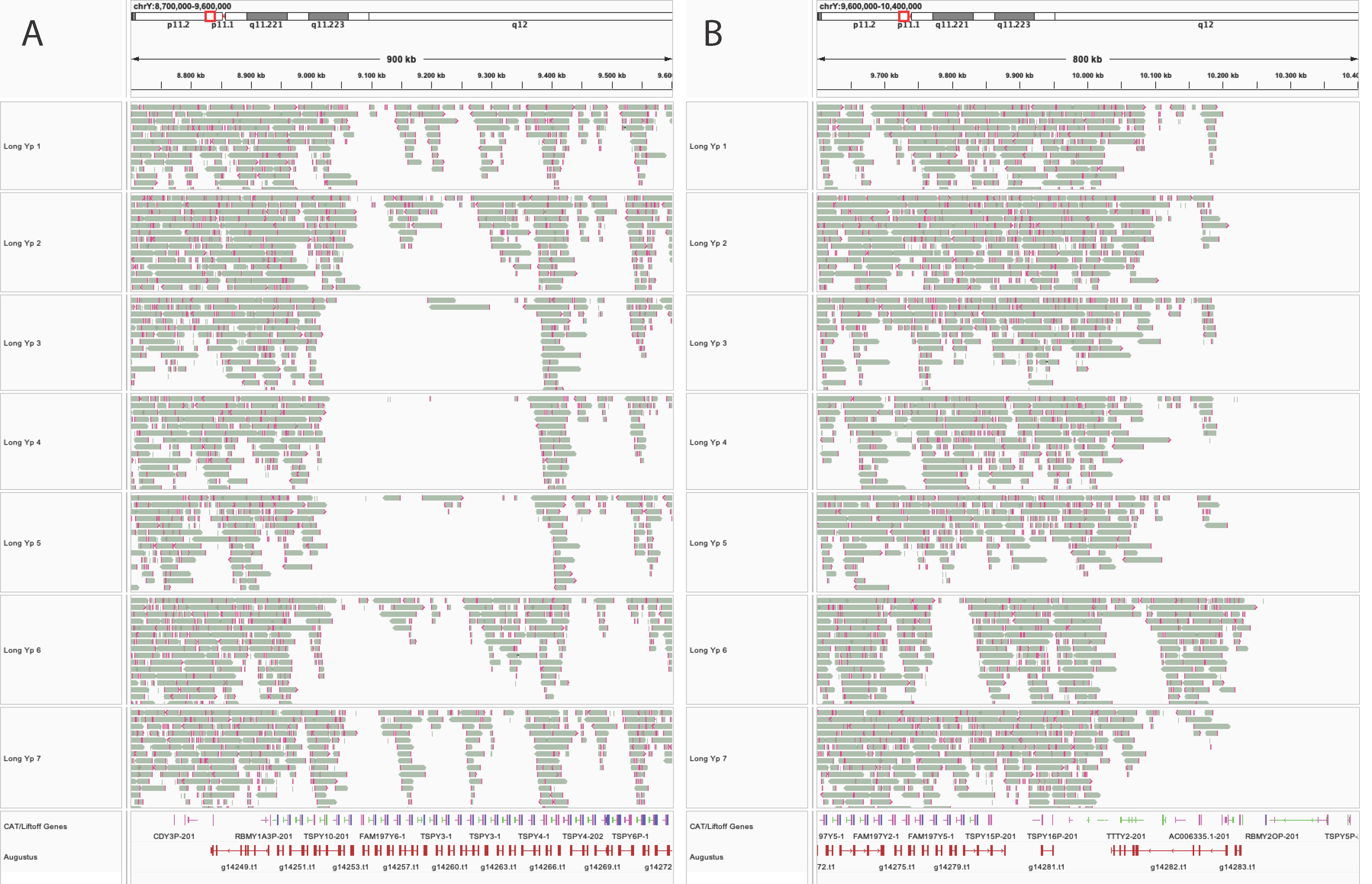

Supplement: Supplementary file 5 — Supplementary Material 5: Supplemental Fig. 2 Incomplete alignment in highly repeated region near the centromere of the Y chromosome. A-B) The seven 46,XX DSD with longer Y chromosome segments (long Yp) had incomplete alignment in the region of the Y chromosome containing highly repeated sequences, including TSPY-genes and FAM197Y-genes. Reads continue to map to the Y chromosome until shortly before the centromere (B). Picture from The Integrative Genomics Viewer (IGV). [file 13293_2024_654_MOESM5_ESM.jpg]

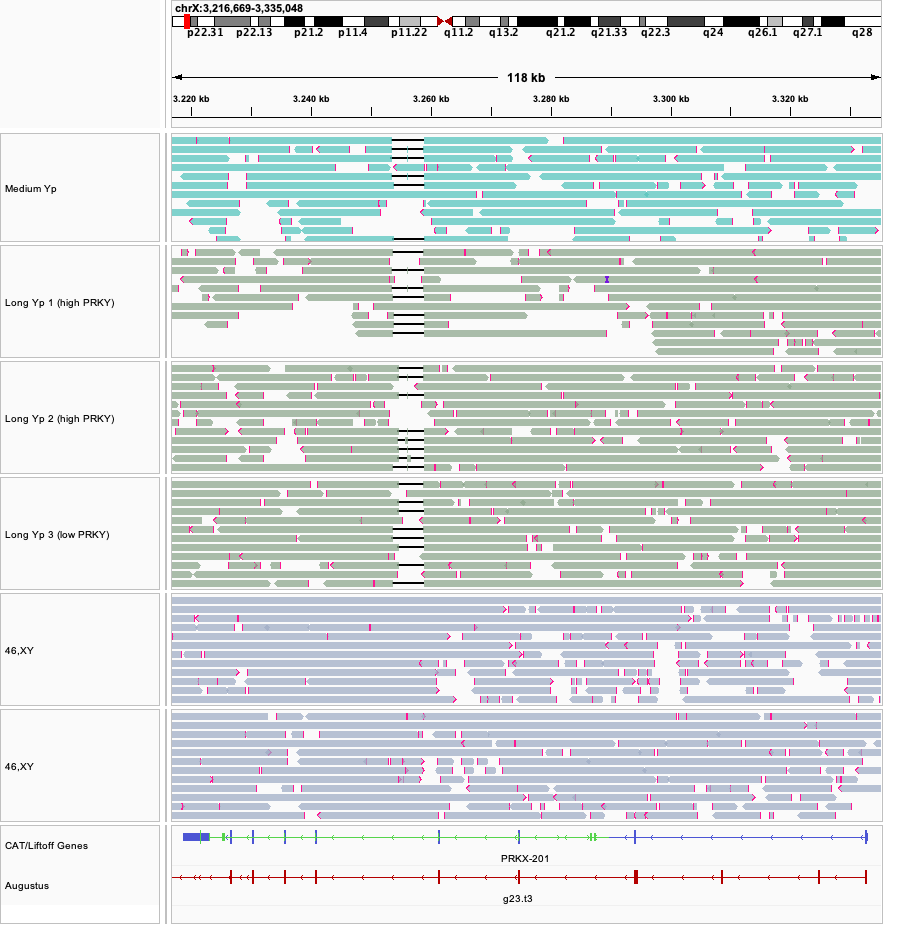

Supplement: Supplementary file 6 — Supplementary Material 6: Supplemental Fig. 3 Partial deletion in PRKX. A partial deletion in PRKY is found in 46,XX DSD (medium Yp, panel 1; representative long Yp, panel 2–4). Two representative 46,XY controls are presented in panel 5–6. Picture from The Integrative Genomics Viewer (IGV). [file 13293_2024_654_MOESM6_ESM.png]

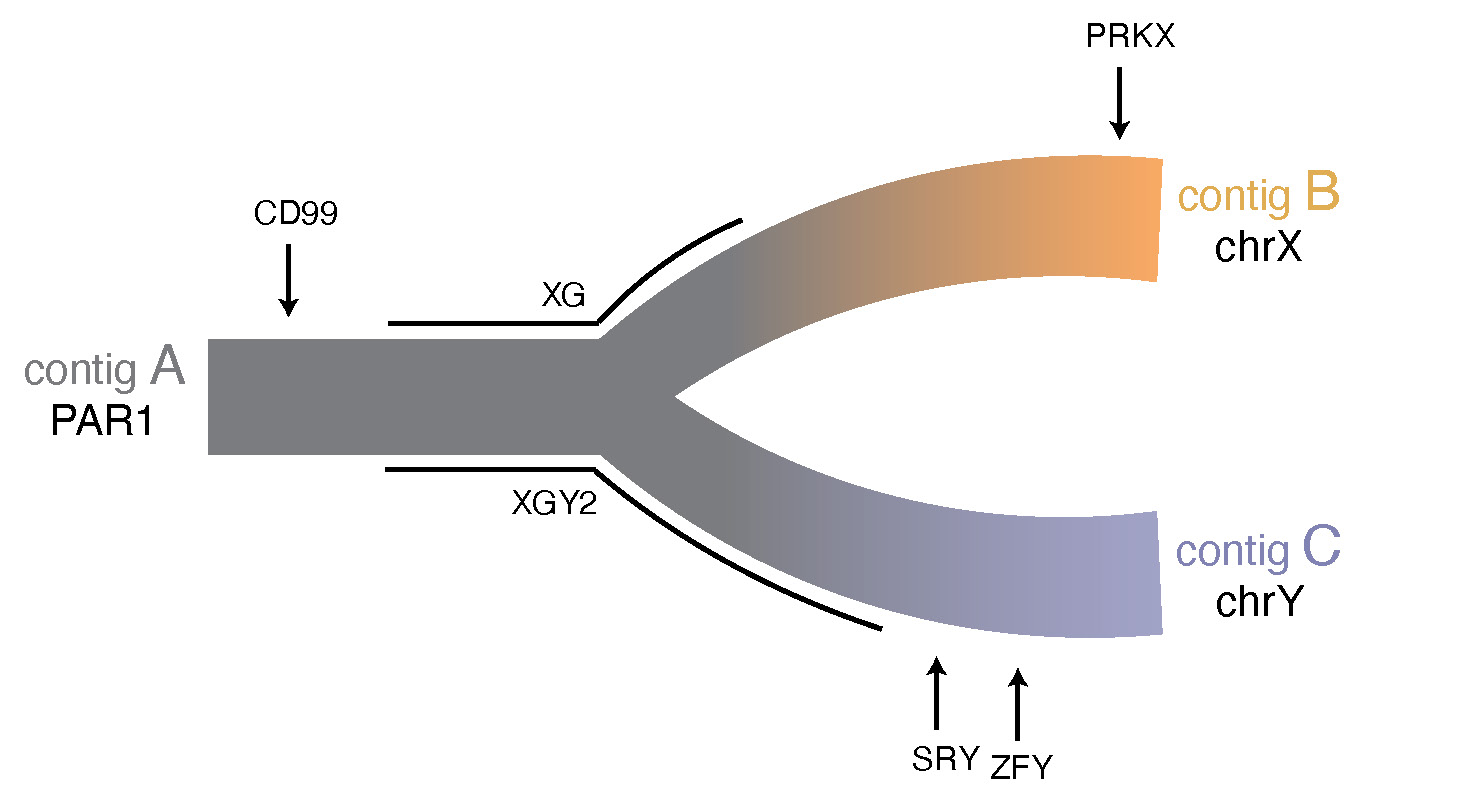

Supplement: Supplementary file 7 — Supplementary Material 7: Supplemental Fig. 4 Parentage of PAR1. A representative illustration of the Y chromosome segment in relation to PAR1 in SRY-positive 46,XX DSD based on de novo assembly and sequence alignments of the assembled contigs indicated that PAR1 is of parental origin. [file 13293_2024_654_MOESM7_ESM.jpg]

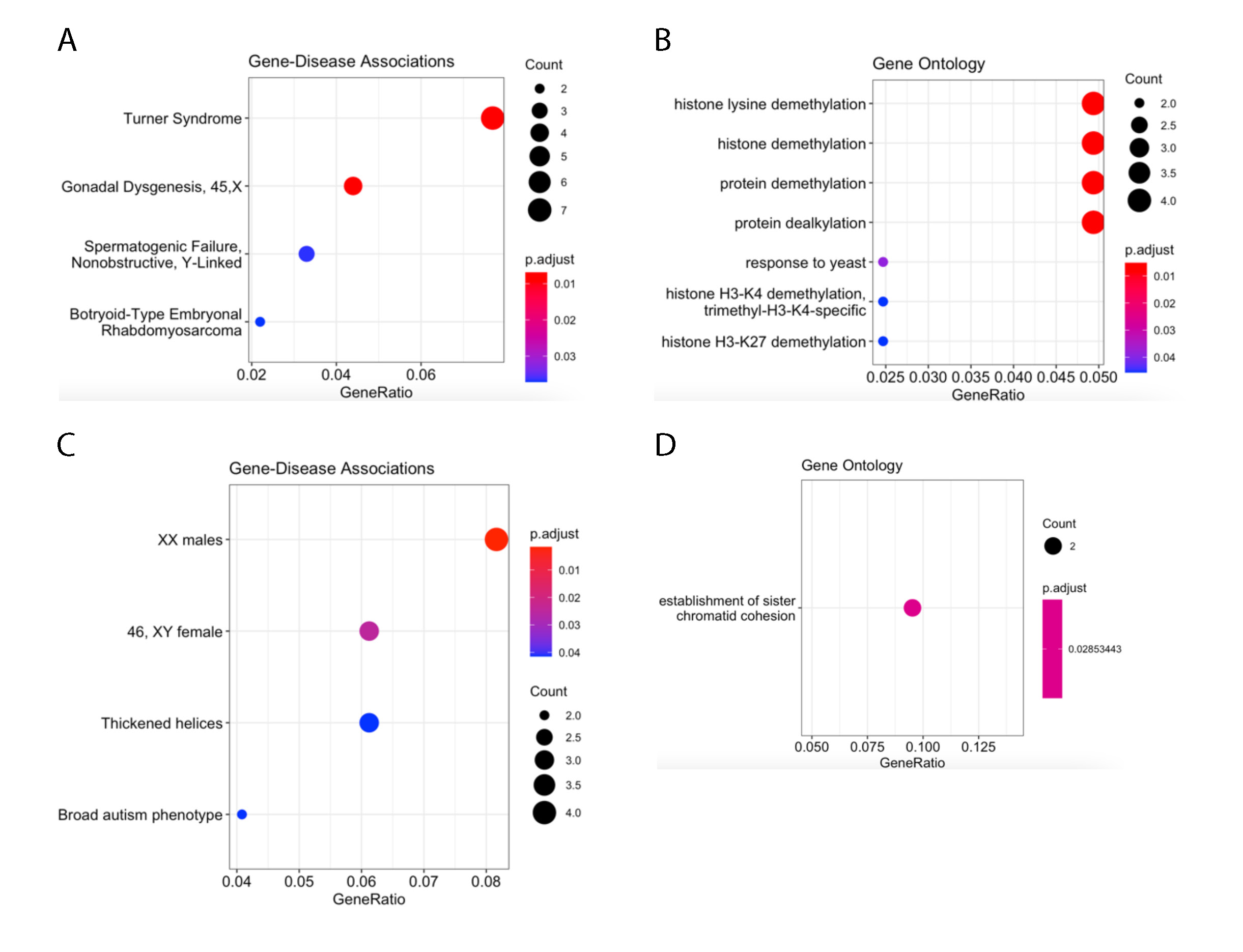

Supplement: Supplementary file 8 — Supplementary Material 8: Supplemental Fig. 5 Differential expression analysis and gene enrichment analysis. Differentially expressed genes in the contrasts. 46,XX DSD vs 46,XX and 46,XX DSD vs 46,XY were used as input for gene enrichment analysis, respectively (Gene-Disease associations, Gene Ontology Biological processes (GOBP). DEGs from the 46,XX DSD vs 46,XY comparison were enriched in diseases related to disorders/differences of sex development and enriched in biological processes involved in methylation (A,B). DEGs from the 46,XX DSD vs 46,XX comparison were also enriched in diseases related to disorders/differences of sex development and enriched in one biological process related to the establishment of sister chromatids (C,D). [file 13293_2024_654_MOESM8_ESM.jpg]

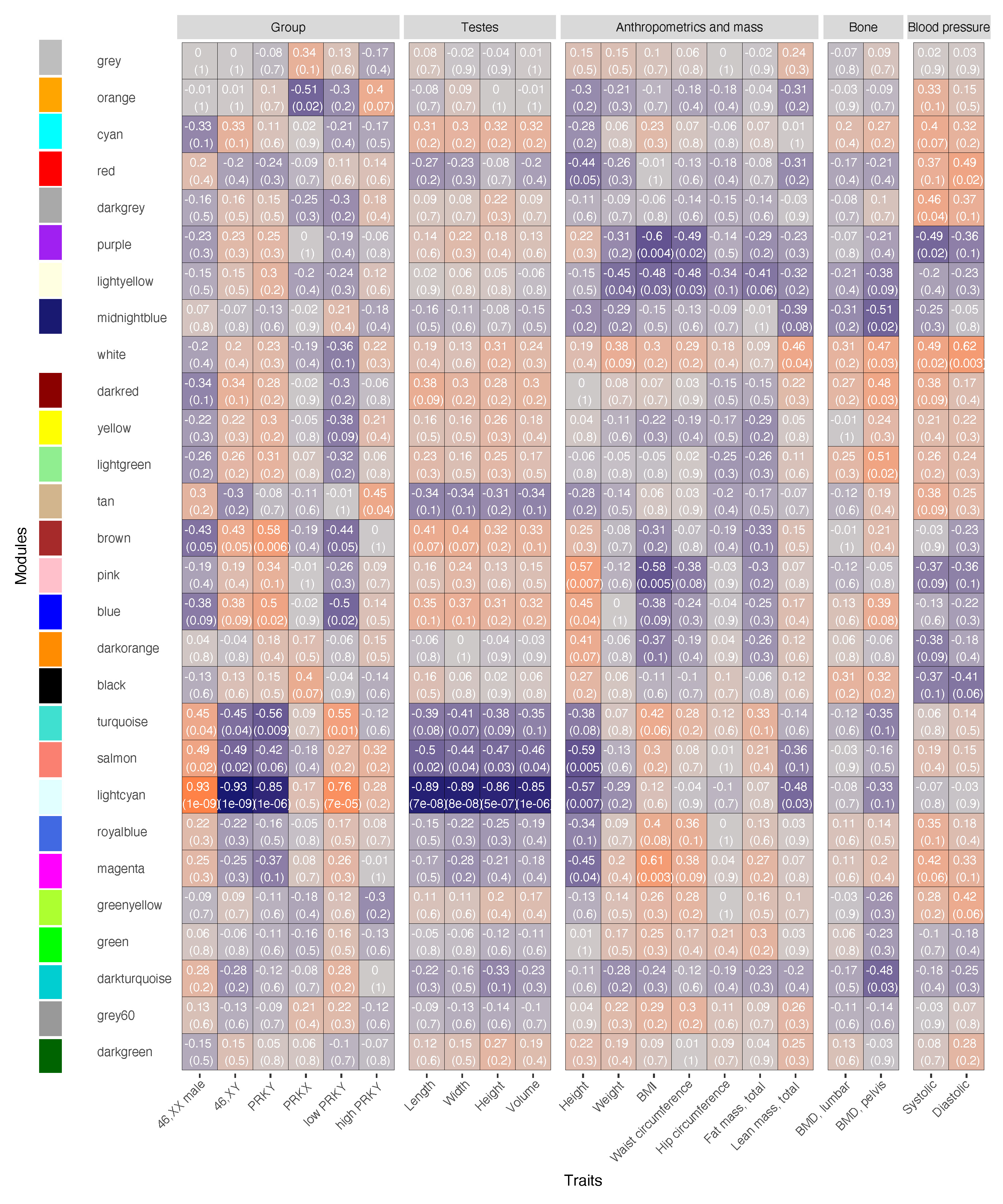

Supplement: Supplementary file 9 — Supplementary Material 9: Supplemental Fig. 6 Weighted Correlation Network Analysis (WGCNA). With gene expression from 11 46,XX DSD and 10 46,XY males as input, a Weighted Correlation Network Analysis (WGCNA) was run to identify modules of co-expressed genes and relate these to the following traits; Patient groups, testis measurements, anthropometrics & mass, bone and blood pressure. Correlation and p-values are shown for each module-trait correlation. [file 13293_2024_654_MOESM9_ESM.jpg]
